# Association of epicardial adipose tissue volume with heart weight in post-mortem cases

**DOI:** 10.1007/s12024-024-00788-6

**Published:** 2024-05-07

**Authors:** Hamish M. Aitken-Buck, Matthew K. Moore, Kyra T. Bingham, Sean Coffey, Rexson D. Tse, Regis R. Lamberts

**Affiliations:** 1 Department of Physiology, HeartOtago, School of Biomedical Sciences, University of Otago, Dunedin, 9054 New Zealand; 2 Department of Medicine, HeartOtago, Dunedin School of Medicine, University of Otago, Dunedin, New Zealand; 3 Department of Cardiology, Dunedin Hospital, Te Whatu Ora, Dunedin, New Zealand; 4 Department of Forensic Pathology, LabPLUS, Auckland City Hospital, Auckland, New Zealand; 5 Griffith University School of Medicine, Southport, QLD Australia; 6 Queensland Public Health and Scientific Services, Coopers Plains, QLD Australia

**Keywords:** Epicardial adipose tissue, Epicardial fat, Heart weight, Heart mass, Post-mortem computed tomography

## Abstract

Epicardial adipose tissue (EAT) deposition has been long associated with heart weight. However, recent research has failed to replicate this association. We aimed to determine the association of EAT volume with heart weight in post-mortem cases and identify potential confounding variables. EAT volume derived from post-mortem computed tomography (PMCT) and heart weight were measured in post-mortem cases (*N* = 87, age: 56 ± 16 years, 28% female). Cases with hypertrophied heart weights (*N* = 44) were determined from reference tables. Univariable associations were tested using Spearman correlation and simple linear regression. Independence was determined with stepwise regression. In the total cohort, EAT volume (median 66 ± 45 cm^3^) was positively associated with heart weight (median 435 ± 132 g) at the univariable level (*r* = 0.6, *P* < 0.0001) and after adjustment for age, female sex, and various body size metrics (*R*^2^ adjusted = 0.41–0.57). Median EAT volume was 1.9-fold greater in cases with hypertrophic hearts (*P* < 0.0001) but with considerably greater variability, especially in cases with extreme EAT volume or heart weight. As such, EAT volume was not associated with heart weight in hypertrophic cases, while a robust independent association was found in non-hypertrophic cases (*R*^2^ adjusted = 0.62–0.86). EAT mass estimated from EAT volume found that EAT comprised approximately 13% of overall heart mass in the total cases. This was significantly greater in cases with hypertrophy (median 15.5%; range, 3.6–36.6%) relative to non-hypertrophied cases (12.5%, 3.3–24.3%) (*P* = 0.04). EAT volume is independently and positively associated with heart weight in post-mortem cases. Excessive heart weight significantly confounded this association.

**Supplementary Information:**

The online version contains supplementary material available at 10.1007/s12024-024-00788-6.

## Introduction

Epicardial adipose tissue (EAT) is the visceral adipose depot of the heart situated between the myocardium and the serous pericardium [1]. For decades, it has been accepted that the amount of EAT deposited around the heart is related to the size of the heart [1, 2]. Plainly, a larger heart has a greater deposition of EAT. Recent research, however, has failed to replicate the positive correlation between EAT deposition and heart weight [3], necessitating this relationship to be re-examined.

Forensic studies have found positive associations between EAT deposition and unindexed heart weight [2, 4, 5]. This is consistent across different modalities of EAT measurement, including qualitative approximation of EAT coverage, dissected EAT weight, and the thickness and area of EAT [2, 4, 5]. Analogous findings have been reported in studies of clinical populations undergoing non-invasive assessment of EAT deposition and myocardial mass. EAT thickness determined from echocardiography has been positively associated with estimates of atrial size, and unindexed and indexed estimates of left ventricle mass [6, 7]. Similarly, EAT volume determined from computed tomography (CT) or cardiac magnetic resonance imaging has been associated with estimates of atrial size and ventricular mass, independent of classic predictors like age, sex, and body size indices [8–13].

Using a semi-quantitative EAT scoring system on transplant hearts, a recent study by Pesczkowski et al. has found different results [3]. With their scoring system, which attempted to incorporate the extent of EAT distribution and observed EAT thickness, they found no relationship between EAT deposition and heart weight in non-ischemic and ischemic hearts [3]. Moreover, they found no association between EAT deposition and well-established predictors like body mass index (BMI) and overall body mass [3, 14]. Due to the growing interest in EAT in pre-clinical and clinical fields, it is prudent to attempt to replicate or provide explanations for the contrary findings.

Hence, for our current study, we aimed to determine the relationship between EAT volume, measured from post-mortem CT (PMCT), and absolute heart weight in a mixed-sex sample post-mortem cohort with diverse EAT volumes and heart weights. This approach allows combining contemporary non-invasive measures of EAT that are limited in post-mortem studies with the absolute heart weight measurements unavailable in clinical imaging studies. A similar approach has previously been used by others to effectively assess the relationships of EAT volume and heart weight in post-mortem cases with coronary artery disease [4].

## Methods

### Post-mortem cases

Adipose volumes and heart weight were measured as part of routine coronial post-mortem examination of 87 consecutive adult cases across 6 months at Auckland City Hospital, Auckland, New Zealand. We have studied the EAT and heart weights of this post-mortem case cohort several times previously [15–18]. The study was approved by the Chief Coroner. Provided that no individuals could be identified by information provided by the forensic pathologist, the University of Otago Human Ethics Committee deemed full ethical review was not required for this study. Post-mortem cases were excluded from this study if the heart was adherent to the surrounding serosa, contained a mechanical valve, or was anatomically compromised (e.g., post-surgical, decomposed, or damaged hearts). No pediatric cases or cases with medico-legal significance were examined for this study. The sex, age, body height, and body weight (measured using inextensible tapes and calibrated scales) were available for each post-mortem case included in the study. From the body height and weight, the BMI (body weight [kg]/body height [m]^2^), body surface area (BSA, DuBois & DuBois formula: (weight [kg]^0.425^ × height [cm]^0.725^) × 0.007284), and estimated fat-free mass (FFM, males: ((height [cm]/100)^1.14^) × (weight [kg]^0.41^)) × 5.1), females: ((height [cm]/100)^1.47^) × (weight [kg]^0.33^)) × 5.34))^19^ were calculated [19]. Post-mortem cases were categorized as non-hypertrophic (*N* = 43) or hypertrophic (*N* = 44) according to whether the individual’s heart weight exceeds the upper confidence limit of the prediction model developed by Vanhaebost and colleagues [20]. The model estimates heart weight from an individual’s sex, height, and weight. Note that ‘hypertrophic’ is used here as a descriptive term, not as a formal diagnostic term.

### Post-mortem computed tomography

EAT, extra-pericardial adipose tissue (ePAT), and visceral adipose tissue (VAT) volumes were measured using PMCT. For the representative PMCT images of each adipose region and in-depth validation of the measurements, refer to the recent study by our group [16]. All post-mortem cases underwent head and torso non-contrast PMCT scans (Siemens SOMATOM Scope, Healthineers AG, Germany) prior to examination. Measurements were made using open access 3D Slicer software, which interpolated between traced segments [21] (https://www.slicer.org/). Adipose measurements were taken from several axial slices. Voxels with Hounsfield unit values between − 190 and − 30 were selected to constitute adipose tissues, with ‘median’ smoothing function applied (3 × 3 mm kernel size).

To measure EAT, the pericardial sac was manually traced between the first piece of the superior tip of the right atrial appendage and the visualization of the right coronary artery inferior to the atrium. The inferior atrioventricular groove was used as the lower bound if the right coronary artery was not visible inferiorly. ePAT was defined as the adipose outside the pericardial sac, excluding most of the lung and mediastinal adipose. Non-EAT intrathoracic adipose does not carry a consensus classifier; hence, ePAT is used here for clarity [16]. VAT was measured as a single slice at the level of the center of the umbilicus. This measurement was significantly correlated with total visceral abdominal adipose tissue measured across multiple slices (*r* = 0.88) [16]. Estimated EAT mass was derived from the known EAT volume as done previously (EAT volume [cm^3^] × 0.92 [specific weight of adipose, g/cm^3^]) [10].

### Heart weight measurement

Heart weight was measured as described previously by our group using a SW15KM digital scale (A & D Company Ltd., Tokyo, Japan; 6000 g capacity, 5 g error margin) [17, 22, 23]. Briefly, as outlined by the European and American guidelines, hearts were removed from the pericardial sac by cutting the great vessels approximately 30 mm above the semilunar valve and by cutting the pulmonary veins at the pericardial reflection. The vena cavae were cut approximately 20 mm above the point where the crest of the right atrial appendage meets the superior vena cava and at the diaphragm. The heart was then dissected in the short axis method, blood and blood clots removed from the heart chambers, and the heart pad dried before being weighed in keeping with most recent recommendations [22, 23]. Hence, the heart weight includes the weight of the heart chambers, the coronary arteries, small segments of the vascular connections, and the EAT.

### Statistical analysis

Where appropriate, continuous data are presented as mean or median values with range and categorical data as number with percentage. The normality of distribution was assessed using the Shapiro-Wilk test and visually using Q-Q plots. Potential outlier EAT volume and heart weight values were determined by ROUT analysis (*Q* = 1%). Differences between hypertrophic and non-hypertrophic groups were determined using unpaired *t*-tests, Mann-Whitney *U* tests, or Fisher’s exact tests according to data normality. Univariable associations were assessed using Spearman correlation or simple linear regression analyses of raw, untransformed data. The independence of predictor associations with heart weight was tested using stepwise linear regression models (*α* to enter model = 0.05, *α* to leave model = 0.05). *Model 1* comprised post-mortem case age, sex (dichotomous, *female* as reference), and one of body height or weight, BSA, FFM, BMI, EAT volume, ePAT volume, or VAT volume. *Model 2* comprised EAT volume alongside post-mortem case age, sex, and one of the body size measurements or indices. Only variables with *α* < 0.05 were retained in the model and reported in the main text with the model *R*^2^ adjusted value. Full model outcomes including constants and estimates are included in the Tables S1 & S3. All analyses were performed using GraphPad Prism (version 10.0.0, GraphPad Software Inc., USA) or Minitab (Minitab Statistical Software, USA) software. *P* < 0.05 was considered statistically significant for all analyses.

## Results

### Post-mortem case characteristics

The key anthropometric characteristics, heart weight metrics, and adipose volumes for the total post-mortem case cohort cases are shown in Table 1. Causes of death included cardiovascular disease/event (*N* = 45), subarachnoid or subdural hemorrhage (*N* = 4), alcohol/drug toxicity (*N* = 12), hanging (*N* = 7), asphyxiation (*N* = 2), asthma (*N* = 2), gastrointestinal hemorrhage (*N* = 3), sepsis (*N* = 2), diabetic ketoacidosis (*N* = 2), or other (*N* = 8). Further cause of death detail can be found in our previous study of this case cohort [17].

**Table 1:** Post-mortem case characteristics, unindexed heart weight, and unindexed adipose volumes measured from post-mortem computed tomography

**Variable**	**Mean/median**	**Range**
Female (*N*, %)	24	28%
Age (years)	56	18 – 86
Body height (cm)	172	148 – 192
Body weight (kg)	80	34 – 148
BMI (kg/m^2^)	28.0	13.0 – 48.9
BSA (m^2^)	2.0	1.3 – 2.6
FFM (kg)	56	34 – 77
Heart weight (g)	435	215 – 865
EAT volume (cm^3^)	66	12 – 221
EAT mass (g)	61	11 – 204
ePAT volume (cm^3^)	54	2 – 101
VAT volume (cm^3^)	30	4 – 79

Data presented as mean or median value with range (continuous variables) depending on normality of distribution or number with percentage (categorical variables)

*N* = 87

*BMI* body mass index, *BSA* body surface area, *FFM* fat-free mass, *EAT* epicardial adipose tissue, *ePAT* extra-pericardial adipose tissue, *VAT* visceral adipose tissue.

Post-mortem cases had a median age of 56 (range of 18–86 years), 28% were female, and the median BMI was 28.0 kg/m^2^ (range, 13.0–48.9 kg/m^2^), indicating an overall middle-aged, male-dominant, overweight case cohort. The median heart weight was 435 g; however, there was a wide range of heart weights in the cohort (215–865 g). A similar range was found for the EAT volume (range, 12–221 cm^3^; median, 66 cm^3^) measured from PMCT and the derived EAT mass (range, 11–204 g; median, 61 g), as well as the ePAT and VAT volumes. Despite the wide variability in heart weights and EAT volumes, no outlier values were identified by ROUT analysis; all values were maintained in the dataset after outlier analysis.

### Univariable associations of body size metrics and adipose volumes with heart weight

Spearman correlation analysis was used to determine the univariable associations of post-mortem case characteristics with heart weight (see Table 2 for correlation coefficients). Female sex was negatively associated with heart weight, while age had no association. As expected [20], unindexed heart weight was positively and robustly associated with anthropometric metrics, including body height and weight, BMI, BSA, and FFM. EAT volume and EAT mass (derived from EAT volume), as well as ePAT and VAT volumes were also significantly and positively associated with unindexed heart weight (Table 2); although, the strength of the ePAT association was notably less. These univariable data indicate that heart weights measured from our post-mortem cohort are associated with classic anthropometric indices and that EAT deposition is associated with heart weight.

**Table 2:** Univariable correlation analyses for heart weight predictors

Response variable: Heart weight vs	***rho***	***P* value**
Female sex	− 0.36	0.001
Age	− 0.02	0.89
Body height	0.51	< 0.0001
Body weight	0.71	< 0.0001
BSA	0.73	< 0.0001
FFM	0.70	< 0.0001
BMI	0.64	< 0.0001
EAT volume/mass	0.60	< 0.0001
ePAT volume	0.30	0.005
VAT volume	0.50	< 0.0001

Spearman correlation analysis performed using raw (non-transformed) values for total case cohort (*N* = 87)

*BMI* body mass index; *BSA* body surface area; *FFM* fat-free mass; *EAT* epicardial adipose tissue; *ePAT* extra-pericardial adipose tissue; *VAT* visceral adipose tissue

### Multivariable associations of body size metrics and adipose volumes with heart weight

Stepwise linear regression was used to determine whether classic anthropometric indices and different adipose volumes are predictors of unindexed heart weight in our case cohort independent of age and sex (*Model 1*). Each classic body size measurement and adipose volume, including EAT volume, was associated with unindexed heart weight independent of age and sex (*Model 1*, Table 3). Of the classic predictors, BSA and FFM could explain 54% and 53% of total heart weight variation, when modelled alongside age and/or female sex. EAT volume and female sex could explain 35% of heart weight variation, while ePAT and VAT volumes could only explain 17% and 23% of heart weight variation, respectively, when modelled with the female sex (Table 3).

**Table 3:** Stepwise linear regression for predictors of heart weight including EAT volume

**Model**	**Independent variable(s)**	***R***^***2***^***adj.***
(1) Variables: Age, female sex + height / weight / BSA / FFM / BMI / EAT volume / ePAT volume / VAT volume	Body height, age	0.25
	Body weight, age	0.49
	BSA, age	0.54
	FFM, age, female sex	0.53
	BMI, age, female sex	0.44
	EAT volume, female sex	0.35
	ePAT volume, female sex	0.17
	VAT volume, female sex	0.23
(2) Variables: As for Model 1 with inclusion of EAT volume	Body height, EAT volume	0.41
	Body weight, female sex, EAT volume	0.54
	BSA, age, EAT volume	0.57
	FFM, EAT volume	0.55
	BMI, EAT volume	0.50

Regressions modelled from total case cohort (*N* = 87). *Model 1* included post-mortem case age, sex, and one of height, weight, BSA, FFM, BMI, EAT volume, ePAT volume, or VAT volume. *Model 2* included the same body size variables as above (non-adipose volumes) with the inclusion of EAT volume. Equivalent Model 2 analyses for ePAT and VAT volumes are shown in Table S1. To enter model *α* = 0.05, to leave model *α* = 0.05. Only independent predictor variables are included in the final models

*BMI* body mass index, *BSA* body surface area, *FFM* fat-free mass, *EAT* epicardial adipose tissue, *ePAT* extra-pericardial adipose tissue, *VAT* visceral adipose tissue

Next, EAT volume, ePAT volume, or VAT volume were added to each model separately to determine whether their association with heart weight is independent of classic body size predictors (*Model 2*). EAT volume was associated with heart weight independent of body height, body weight, BSA, FFM, BMI, ePAT volume, or VAT volume (Table 3). Moreover, the inclusion of EAT volume improved the *R*^2^ adjusted value for each model. The association of ePAT volume with heart weight was maintained after adjusting for body height and BMI, but not for body weight, BSA, FFM, or EAT volume (Table S1). The association of VAT with heart weight was independent of body height, but not of body weight, BSA, FFM, BMI, or EAT volume (Table S1). Together, these data confirm that EAT volume is associated with heart weight independently of age, sex, classic body size predictors, and other visceral adipose volumes.

### Differential associations with heart weight in cardiac hypertrophy

**Fig. 1:**
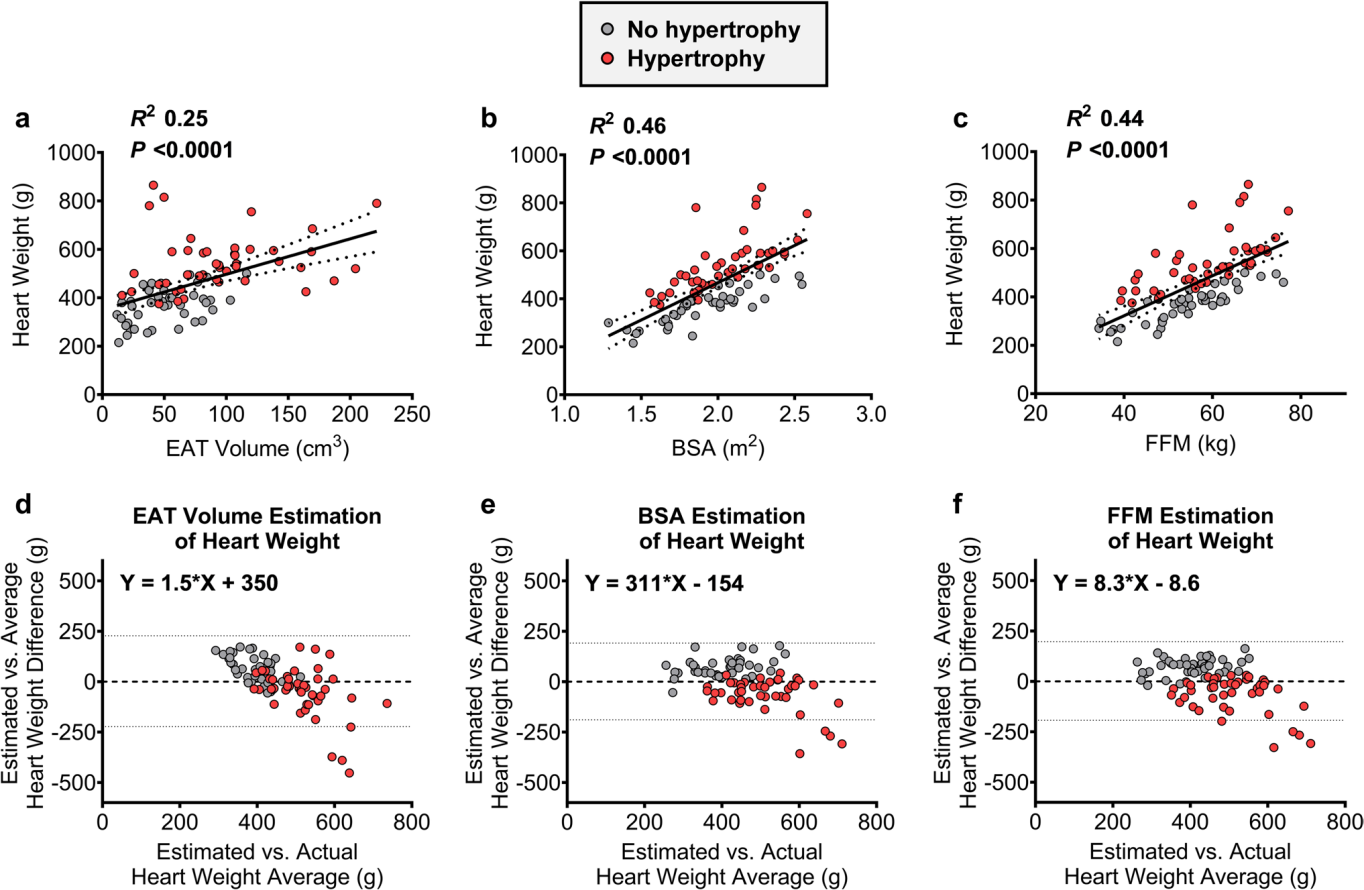
Association and prediction of heart weight with EAT volume, BSA, and FFM. **a**–**c** simple linear regression analyses of EAT volume (**a**), BSA (**b**), and FFM (**c**) as univariable predictors of heart weight. **d**–**f** Bland-Altman plots showing heart weight prediction accuracy of estimates derived from simple linear regressions of the EAT volume (**d**), BSA (**e**), and FFM (**f**) associations with heart weight. All analyses were performed on raw (non-transformed) data. EAT, epicardial adipose tissue; BSA, body surface area; FFM, fat-free mass.* N* = 87

The simple linear regression of EAT volume as a predictor of heart weight can be appreciated in Fig. 1 (*R*^2^ for total cohort = 0.25). However, it becomes apparent that more of the variation in the EAT volume association with heart weight is found in post-mortem cases with large heart weights or EAT volumes. To explore this further, we categorized the cases into those with (median heart weight, 528 g; range, 375–865 g) or without (median, 380 g; range, 215–500 g) cardiac hypertrophy according to contemporary heart weight prediction tools [20]. Relative to the cases without hypertrophy, the cases with hypertrophy, especially those with an extreme heart weight or EAT volume, diverge considerably from the regression line (Fig. 1a). This divergence is observationally greater than that for the heart weight association with classic predictors BSA or FFM (Fig. 1b and c). Limitations in heart weight estimation from a known EAT volume are also observable in the Bland-Altman plot in Fig. 1D, whereby the estimation accuracy is diminished at the extremes of heart weight values. Again, this pattern is more pronounced for EAT volume estimation of heart weight than for estimation from BSA or FFM (Fig. 1e and f).

**Fig. 2:**
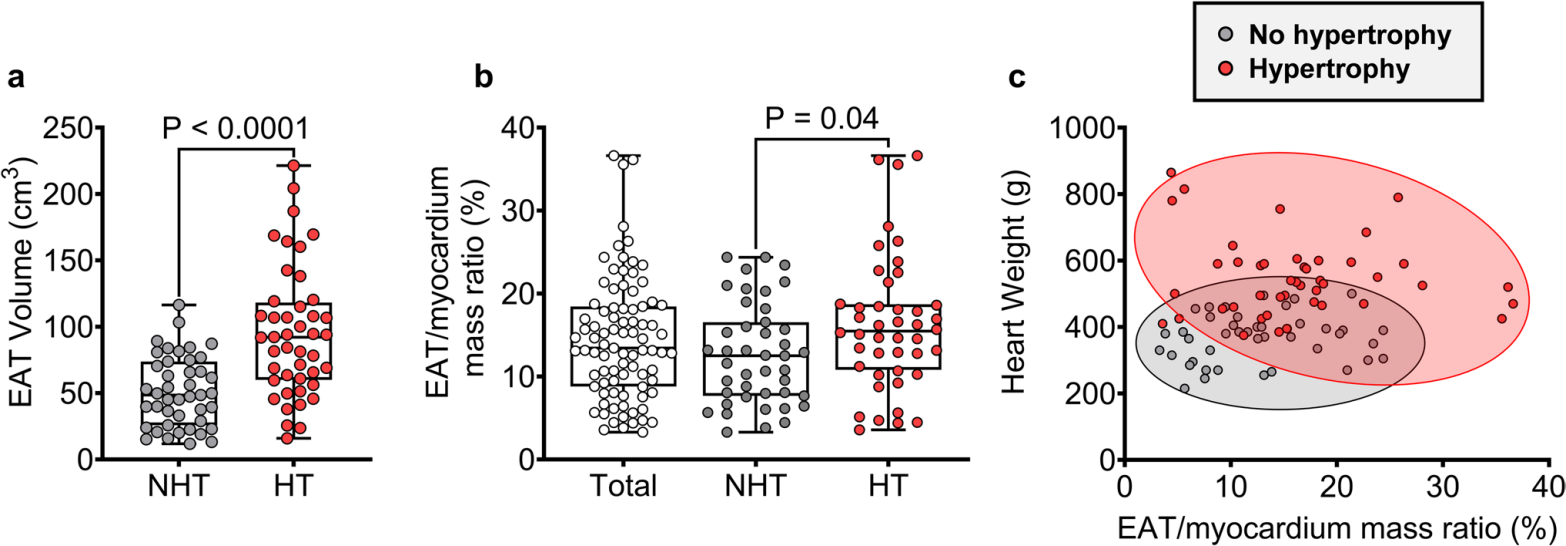
EAT/myocardium mass ratio differences in cardiac hypertrophy. **a** Median EAT/myocardium mass ratios in the total post-mortem cases and cases without hypertrophy (non-hypertrophied, NHT, grey) and with hypertrophy (HT, red). **b** Scatter plot showing association between EAT/myocardium mass ratio and heart weight in NHT and HT groups. For **a**, the difference between NHT and HT groups was determined using the Mann-Whitney *U* test. NHT *N* = 43, HT *N* = 44

The non-hypertrophic and hypertrophic group characteristics are shown in Table S2. There was no difference in the median age or proportion of females. The hypertrophic group had significantly greater median body weight, BMI, BSA, FFM, ePAT volume, and VAT volume (Table S2). The median EAT volume (Fig. 2a) and EAT mass (Table S2) were approximately 1.9-fold greater in the hypertrophic group.

These univariable data indicate that hypertrophy status is an important confounding variable in the EAT volume association with heart weight. We then confirmed this using stepwise linear regression incorporating EAT volume alongside age, female sex, and different body size metrics (Table 4). In each model, EAT volume was independently associated with heart volume. However, this was only present in non-hypertrophied cases. Interestingly, models incorporating EAT volume with BSA or FFM and age or female sex could explain up to 86% of heart weight variation in non-hypertrophied cases.

**Table 4:** Stepwise linear regression for predictors of heart weight in post-mortem cases with and without cardiac hypertrophy

**Model**	***No hypertrophy***	
Variables: Age, female sex + height / weight / BSA / FFM / BMI + EAT volume	Anthropometric variable(s)	*R*^*2*^* adj.*
	Body height, female sex, EAT volume	0.62
	Body weight, age, female sex, EAT volume	0.81
	BSA, age, female sex, EAT volume	0.86
	FFM, age, EAT volume	0.86
	BMI, female sex, EAT volume	0.68
	*Hypertrophy*	
	Anthropometric variable(s)	*R*^*2*^* adj.*
	Body height	0.21
	Body weight	0.41
	BSA	0.43
	FFM	0.40
	BMI, female sex	0.38

To enter model *α* = 0.05, to leave model *α* = 0.05. Only independent predictor variables are included in the final models. Each model included post-mortem case age, sex, EAT volume, and one of height, weight, BSA, FFM, or BMI. No hypertrophy *N* = 43, hypertrophy *N* = 44

*BMI* body mass index, *BSA* body surface area, *FFM* fat-free mass, *EAT* epicardial adipose tissue

### EAT/myocardium mass ratio in non-hypertrophied and hypertrophied hearts

Next, we used EAT mass estimated from the EAT volume to determine if the EAT/myocardium mass ratio differs in hypertrophied hearts. In the total post-mortem case cohort, the median EAT/myocardium mass ratio was 13.4% with a range from 3.3 to 36.6% (Fig. 2b). The median EAT/myocardium mass ratio was significantly greater in hypertrophied hearts (15.5%) relative to non-hypertrophied hearts (12.5%), despite having considerably greater variation (hypertrophied, 3.6–36.6%; non-hypertrophied, 3.3–16.5%). The variation in EAT/myocardium mass ratio for a given heart weight in hypertrophic cases can also be appreciated in the scatter plot in Fig. 2c. Simple regression analysis identified hypertrophy classification as a significant univariable predictor of the EAT/myocardium mass ratio alongside age, ePAT volume, and VAT volume (Table 5). Stepwise linear regression incorporating all variables found that age, ePAT volume, VAT volume, as well as female sex were the only independent predictors (*R*^2^ = 0.45) (Table 5). These data indicate an imbalance in EAT and myocardial composition of the heart in cardiac hypertrophy.

**Table 5:** Regression analyses for predictors of the EAT/myocardium mass ratio in post-mortem cases

Response variable: EAT/myocardium mass ratio	Simple regression			Multiple regression		
	*β*	*P* value	*R*^*2*^	*β*	*P* value	*R*^*2*^* adj.*
Female sex	2.1	0.2	0.02	2.8	0.04	0.45
Age	0.2	< 0.0001	0.17	0.1	0.02	
Pathological hypertrophy	3.6	0.02	0.06			
Body height	− 0.1	0.4	0.01			
Body weight	0.01	0.7	0.02			
BMI	0.09	0.4	0.01			
BSA	0.4	0.9	0.00			
FFM	− 0.0	0.7	0.00			
ePAT volume	0.1	< 0.0001	0.20	0.06	0.009	
VAT volume	0.2	< 0.0001	0.28	0.2	< 0.0001	

Simple linear regression analyses for association with EAT/myocardium mass ratio were performed for each variable. Stepwise linear regression analyses were then performed to determine independent associations. To enter multivariable model *α* = 0.05, to leave model *α* = 0.05. Only independent predictor variables included in the final model. *N* = 87

*EAT* epicardial adipose tissue, *BMI* body mass index, *BSA* body surface area, *FFM* fat-free mass, *ePAT* extra-pericardial adipose tissue, *VAT* visceral adipose tissue

## Discussion

In several previous post-mortem studies, EAT deposition increased in parallel with heart weight [2, 4, 5]. A recent contrary report [3] suggests that this relationship between EAT and the heart weight should be re-investigated. By utilizing EAT volumes measured non-invasively on PMCT and heart weights determined during autopsy, this study shows that EAT volume is strongly and independently associated with heart weight in post-mortem cases. Importantly, this relationship, while present in the total case cohort, was only present in hearts with normal weights, with no association found in hearts classified as hypertrophic. Together, this confirms EAT deposition is indeed associated with heart weight; however, the degree of cardiac hypertrophy influences the relationship.

### Previous studies of EAT deposition and heart weight

Like classic body size predictors (body height and weight, BSA, FFM, and BMI), EAT volume is associated with heart weight in our mixed-sex post-mortem case cohort with diverse heart weights and hypertrophy status (*r* = 0.60). This aligns with previous post-mortem studies with comparable case characteristics and heart weight distributions that identified associations of heart weight with the amount of EAT on the anterior surface of the heart (*r* = 0.68) [4], or with the weight of EAT dissectible from the heart [2, 5]. We have also confirmed that EAT volume is associated with heart weight independent of age, sex, and various body size metrics, which is important given the covariance of EAT volume with these variables [14]. Our results also align with clinical findings of EAT volume (measured on CT scans) positively and independently associating with unindexed left ventricle mass and left ventricle mass indexed to height^2.7^ or BSA [8, 9]. Together, our findings confirm that EAT deposition is associated with heart weight. Physiologically, this is consistent with the hypothesis that EAT provides local support to the metabolic, contractile, and growth needs of the heart [24]. If the heart is required to grow, as occurs naturally in age or in response to exercise, it seems that metabolic support provided by EAT would increase in parallel, thus resulting in greater EAT deposition. Further longitudinal work either in humans or large animal models that natively develop EAT is required to confirm that EAT deposition is necessary to enable the heart to grow in response to physiological or pathophysiological stimuli.

### EAT relationship with cardiac hypertrophy

Our study revealed that cardiac hypertrophy was an important confounding variable in the EAT volume association with heart weight. In line with previous findings of Corradi et al. [2], median EAT volume was 1.9-fold greater in cases with hearts classified as hypertrophic by contemporary heart size estimation calculators [20]. However, despite having a greater amount of EAT, the association of EAT volume with heart weight identified in our total case cohort was absent in hypertrophic cases. Conversely, in non-hypertrophic hearts, this relationship was maintained. Therefore, an overrepresentation of hypertrophic hearts in studies of the EAT deposition relationship with heart weight would diminish or remove this association. This would explain the recent contrary findings that partly inspired this study [3]. Although not stated, most heart weights used in the contrary study appear to be greater than 400 g, with a pronounced cluster between 500 and 600 g [3]. This would place the hearts used in their analyses within hypertrophic groups if categorized using post-mortem heart weight estimation calculators. Therefore, it is likely that no association between their semi-quantitative measure of EAT and transplant heart weight was found due to sample selection and the confounding effect of cardiac hypertrophy.

### EAT comprises a greater proportion of heart weight in hypertrophy

By calculating EAT mass from EAT volume [10], we also found that EAT comprises approximately 13.3% of the heart weight in our total case cohort. This is congruent with previous estimates of EAT comprising 14.7% of total heart weight in post-mortem cases with mixed hypertrophy status [2]. In non-hypertrophic cases, EAT comprised 12.5% of total heart weight, indicating that EAT constitutes approximately one-eighth of all tissue mass in normal hearts. In contrast, hypertrophic hearts were made up of 15.5% EAT, with several cases having EAT ratios exceeding 30% of total heart weight. Despite the increased relative abundance of EAT in hypertrophic hearts, hypertrophy status was not associated with the EAT/myocardium mass ratio in simple linear regression or multivariable regression (adjusted for variables including age, female sex, and other adiposity measures). This lack of association is likely due to the high degree of variability of EAT/myocardium mass ratios in the hypertrophic group in combination with the broad range of heart weights classified as hypertrophic using post-mortem estimators. Moreover, no information was available from our cohort regarding the underlying cause or stage of hypertrophy. We suspect a more refined classification criteria and known cause or stage of hypertrophy would reveal hypertrophy as a better predictor of the EAT/myocardium mass ratio.

These findings establish a rationale for not only clinical and post-mortem studies of the EAT/myocardium mass ratio as a diagnostic tool but also for pre-clinical studies of what drives the disproportionate deposition of EAT. Furthermore, they warrant investigation of how the relative excess of EAT in the heart composition affects the metabolic and paracrine signaling roles of EAT.

### Comparison to other adipose volumes

We performed the same univariable and multivariable analyses of ePAT and VAT volumes as predictors of heart weight. Both were associated with heart weight univariably and after age and sex adjustment but not after BSA or FFM adjustment. This supports that heart weight is more accurately predicted by indices of body size and muscle mass rather than measures of non-cardiac adiposity [25]. Unlike the classic anthropometric predictors of heart weight, ePAT, and VAT volumes were independently associated with the EAT/myocardium mass ratio. This suggests that these measures of visceral adiposity have greater utility in estimating potentially pathological deposition of EAT rather than predicting heart weight. Since ePAT and VAT volumes were not predictive of heart weight, our results also indicate that EAT volume more closely resembles body size metrics as heart weight predictors rather than adiposity metrics or other adipose volumes. This further supports the concept that nourishment provided by EAT to the myocardium is required for the homeostasis and growth of the heart [24]. Additionally, it highlights the potential utility in incorporating EAT volume into post-mortem heart mass estimation models. Our regression models indicate that including EAT volume with BSA or FFM and age, up to 86% of non-hypertrophic heart weight variability can be explained.

### Limitations

Several limitations should be considered alongside these findings. Firstly, despite allowing assessment of absolute heart weight, the information available for each post-mortem case was limited to age, sex, and body height and weight. EAT deposition is affected by exercise, dietary intervention, and several pharmaceutical drugs, including thiazolidinediones, glucagon-like peptide 1 receptor agonists, and statins [26]. Hence, without being able to incorporate these variables into our prediction models, we cannot rule out their potential influence on the EAT volume association with heart weight. Secondly, we have shown here that age is a univariable and multivariable (depending on the model) predictor of heart weight and the EAT/myocardium mass ratio. Therefore, when extrapolating the findings to clinical patient cohorts with cardiovascular disease, it is also important to consider the younger average age and broader age range of post-mortem case cohorts. Thirdly, the EAT volumes measured for this study were from post-mortem cases. A certain degree of decomposition of adipose tissues, and in particular inconsistent decomposition between cases, therefore, cannot be definitively ruled out as a confounding factor despite only including non-decomposed cases in the study. Sedimentation of blood also occurs in the post-mortem period [16], which could potentially manifest as artifacts on PMCT images. Importantly, the Hounsfield unit ranges of adipose tissue and sedimented fluid do not overlap; therefore, we are confident this has not led to EAT volume measurement error. Finally, we have used total EAT volume as the predictor of interest for this study. However, EAT tends to accumulate in specific foci like the anterior surface of the right ventricle [1]. Accumulation of EAT in specific heart regions could associate differently with heart weight or confer different levels of disease risk, like that reported previously [4]. Therefore, a follow-up research should consider the regionality of EAT distribution.

## Conclusion

EAT volume measured with PMCT was positively associated with heart weight independent of age, sex, and various classic body size metrics. While this relationship was present in the total case cohort with diversity of heart weights, the association was robust in cases without cardiac hypertrophy, but absent in cases with cardiac hypertrophy. These findings confirm that individuals with normal heart weights have predictable EAT deposition, as established previously [2, 4, 5], while also identifying cardiac hypertrophy as an important confounding variable. This provides a clear rationale for future pre-clinical and clinical research into how the imbalance in EAT deposition in cardiac hypertrophy impacts myocardial metabolism and disease pathogenesis and progression.

## Key points

- The long-standing positive association between EAT volume and heart weight has recently been questioned. This study aimed to re-examine this association in a cohort of post-mortem cases with diverse heart weights.
- In the total post-mortem cohort, EAT comprised approximately 13% of overall heart weight, and EAT volume was positively associated with heart weight independent of age, female sex, and various body size metrics.
- Cases with heart weights within normal reference limits had robust and independent positive associations of EAT volume with heart weight.
- Conversely, despite having significantly greater median EAT volume and EAT/myocardium mass ratio than non-hypertrophic cases, cases with significant cardiac hypertrophy had no relationship between EAT volume and heart weight.
- These findings confirm that EAT volume and heart weight increase proportionally. However, this relationship is only present in individuals with normal heart weights; cardiac hypertrophy, especially extreme hypertrophy, confounds this relationship.

## Supplementary Information

 Below is the link to the electronic supplementary material.

Supplementary file1 (DOCX 23.1 KB)

## Data Availability

Data supporting the findings of this study are available from the corresponding author [RRL] on request.
