# Supplementary material for: Association of epicardial adipose tissue volume with heart weight in post-mortem cases
Source: Forensic Sci Med Pathol. 2024 May 7;20(4):1251–60. doi: 10.1007/s12024-024-00788-6 (PMC11790810; doi:10.1007/s12024-024-00788-6)
Supplement: Supplementary file 1 — Supplementary file1 (DOCX 23.1 KB) [file 12024_2024_788_MOESM1_ESM.docx]

**Supplementary Material**

***Forensic Science, Medicine and Pathology***

**Association of epicardial adipose tissue volume with heart weight in post-mortem cases**

| **Total post-mortem cases (*N* = 87).** Response variable: heart weight | | | |
| --- | --- | --- | --- |
| **Model** | **Predictor** | **Regression equation** | ***R^2^ adj.*** |
| 1 | Body height | -1051 + 1.83 (age) + 8.2 (height) | 0.25 |
| 2 | + EAT volume | -725 + 6.3 (height) + 1.3 (EAT) | 0.41 |
| 2 | + ePAT volume | -734 + 6.6 (height) + 1.0 (ePAT) | 0.26 |
| 2 | + VAT volume | -738 + 6.5 (height) + 2.5 (VAT) | 0.32 |
| 1 | Body weight | 16 + 2.4 (age) + 4.0 (weight) | 0.49 |
| 2 | + EAT volume | 178 + 2.6 (weight) + 1.0 (EAT) | 0.54 |
| 2 | + ePAT volume | -16 + 2.4 (age) + 4.0 (weight) | 0.50 |
| 2 | + VAT volume | -16 + 2.4 (age) + 4.0 (weight) | 0.50 |
| 1 | BMI | 60 + 1.9 (age) + 10.9 (BMI) – 82 (female) | 0.44 |
| 2 | + EAT volume | 182 + 7.8 (BMI) + 1.0 (EAT) – 78 (female) | 0.50 |
| 2 | + ePAT volume | 126 + 10.0 (BMI) + 1.2 (ePAT) – 71 (female) | 0.46 |
| 2 | + VAT volume | 60 + 1.9 (age) + 10.9 (BMI) – 82 (female) | 0.44 |
| 1 | BSA | -398 + 2.6 (age) + 360 (BSA) | 0.54 |
| 2 | + EAT volume | -294 + 1.7 (age) + 311 (BSA) + 0.6 (EAT) | 0.57 |
| 2 | + ePAT volume | -398 + 2.6 (age) + 360 (BSA) | 0.54 |
| 2 | + VAT volume | -398 + 2.6 (age) + 360 (BSA) | 0.54 |
| 1 | FFM | -366 + 2.6 (age) + 11.7 (FFM) + 74 (female) | 0.53 |
| 2 | + EAT volume | -22 + 7.2 (FFM) + 1.0 (EAT) | 0.55 |
| 2 | + ePAT volume | -366 + 2.6 (age) + 11.7 (FFM) + 74 (female) | 0.53 |
| 2 | + VAT volume | -366 + 2.6 (age) + 11.7 (FFM) + 74 (female) | 0.53 |
| 1 | EAT volume | 381 + 1.4 (EAT) -98 (female) | 0.35 |
| 1 | ePAT volume | 421 + 1.2 (ePAT) – 98 (female) | 0.17 |
| 2 | + EAT volume | 381 + 1.4 (EAT) -98 (female) | 0.35 |
| 1 | VAT volume | 398 + 2.7 (VAT) – 94 (female) | 0.23 |
| 2 | + EAT volume | 381 + 1.4 (EAT) -98 (female) | 0.35 |

**Table S1**. **Full model equations from stepwise linear regressions for predictors of heart weight**. Regressions modelled from total case cohort (*N*=87). *Model 1* included post-mortem case age, sex, and one of height, weight, BSA, FFM, BMI, EAT volume, ePAT volume, or VAT volume. *Model 2* included the same body size variables as above (non-adipose volumes) with inclusion of EAT volume. To enter model α = 0.05, to leave model α = 0.05. Only independent predictor variables are included in the final models. BMI = body mass index; BSA = body surface area; FFM = fat-free mass; EAT = epicardial adipose tissue; ePAT = extra-pericardial adipose tissue; VAT = visceral adipose tissue.

|  | **No Hypertrophy**  (*n*=43) | | **Hypertrophy**  (*n*=44) | |  |
| --- | --- | --- | --- | --- | --- |
| **Variable** | **Median** | **Range** | **Median** | **Range** | ***P*** |
| Age (years) | 55 | 18 - 81 | 58 | 31 - 86 | 0.05 |
| Female (n, %) | 13 | 30% | 11 | 25% | 0.64 |
| Body height (cm) | 171 | 148 - 190 | 174 | 155 - 192 | 0.24 |
| Body weight (kg) | 75 | 34 - 148 | 90 | 55 - 145 | 0.009 |
| BMI (kg/m^2^) | 26.0 | 13.0 - 48.9 | 30.0 | 19.0 - 47.4 | 0.007 |
| BSA (m^2^) | 1.9 | 1.3 - 2.6 | 2.0 | 1.6 - 2.6 | 0.01 |
| FFM (kg) | 55 | 34 - 76 | 59 | 39 - 77 | 0.03 |
| Heart weight (g) | 380 | 215 - 500 | 528 | 375 - 865 | <0.0001 |
| EAT mass (g) | 45 | 11 - 107 | 85 | 15 - 204 | <0.0001 |
| ePAT volume (cm^3^) | 43 | 2 - 97 | 67 | 8 - 101 | 0.002 |
| VAT (cm^3^) | 23 | 4 - 79 | 38 | 4 - 72 | 0.0004 |

**Table S2**. **Post-mortem case characteristics, unindexed heart weights, and adipose volumes measured from post-mortem computed tomography of cases without hypertrophy and with hypertrophy**. Data presented as median value with range (continuous variables) or number with percentage (categorical variables). No hypertrophy vs. hypertrophy differences determined using Mann-Whitney U-tests (body weight, heart weight, EAT mass, extra-pericardial fat volume, visceral fat volume) or unpaired t-tests (age, body height, BSA, FFM) according to normality of distribution, or Fisher’s exact test (female sex proportion). BMI = body mass index; BSA = body surface area; FFM = fat-free mass; EAT = epicardial adipose tissue.

| **Non-hypertrophic and hypertrophic post-mortem cases.** Response variable: heart weight. | | | |
| --- | --- | --- | --- |
| **Predictor** | **Group** | **Regression equation** | ***R^2^ adj.*** |
| Body height + EAT volume | NH | -340 + 3.9 (height) + 1.2 (EAT) -38 (female) | 0.62 |
|  | H | -636 + 6.9 (height) | 0.21 |
| Body weight + EAT volume | NH | 133 + 0.9 (age) + 2.1 (weight) + 0.6 (EAT) – 45 female) | 0.81 |
|  | H | 251 + 3.2 (weight) | 0.41 |
| BMI  + EAT volume | NH | 200 + 5.2 (BMI) + 0.9 (EAT) – 63 (female) | 0.68 |
|  | H | 302 + 8.7 (BMI) – 85 (female) | 0.38 |
| BSA  + EAT volume | NH | -78 + 1.0 (age) + 197 (BSA) + 0.6 (EAT) – 30 (female) | 0.86 |
|  | H | -52 + 292 (BSA) | 0.43 |
| FFM  + EAT volume | NH | -37 + 0.8 (age) + 6.1 (FFM) + 0.7 (EAT) | 0.86 |
|  | H | 123 + 7.2 (FFM) | 0.40 |

**Table S2**. **Stepwise linear regression for predictors of heart weight in** **post-mortem cases without hypertrophy (no hypertrophy, NH) and with hypertrophy (HT)**. To enter model α = 0.05, to leave model α = 0.05. Only independent predictor variables included in the final model. Each model included post-mortem case age, sex, EAT volume, and one of height, weight, BSA, FFM, or BMI. BMI = body mass index; BSA = body surface area; FFM = fat-free mass; EAT = epicardial adipose tissue. No hypertrophy *n* = 43, hypertrophy *n* = 44.
